# Factors influencing gender preference towards surgeons among Jordanian adults: an investigation of healthcare bias

**DOI:** 10.1038/s41598-023-38734-1

**Published:** 2023-07-18

**Authors:** Jehad Feras AlSamhori, Rama Rayyan, Muhammad Hammouri, Hana Taha, Leen Al-Huneidy, Wahid AlOweiwi, Jamil AlMohtasib, Shahd Mansour, Majid Dardas, Jamil Qiqieh, Zeina Halasa, Yazan Al-Huneidy, Abdallah Al-Ani

**Affiliations:** 1 School of Medicine, The University of Jordan, Amman, Jordan; 2 Department of Pharmacology, Public Health and Clinical Skills, Faculty of Medicine, The Hashemite University, P.O. Box 330127, Zarqa, 13133 Jordan; 3 Department of Internal Medicine, King Hussein Medical Center, Amman, Jordan; 4 Office of Scientific Affairs and Research, King Hussein Cancer Center, Amman, Jordan

**Keywords:** Health policy, Health occupations

## Abstract

Studies investigating gender bias against female surgeons yielded conflicting results ranging from neutrality to a clear preference towards male surgeons. Yet, such bias remains understudied within Middle Eastern nations. We aimed to assess preferences of surgeons’ gender among Jordanians and explore reasons for possible gender bias across different surgical specialties. A total of 1708 respondents were examined using a cross-sectional, self-administered questionnaire to evaluate the gender preferences of surgeons, characteristics associated with preferred surgeon’s gender, and surgeon’s preference in certain specialties. Nearly 52.0% of participants had no gender preference for surgeons. Among those with a preference, 75.7% preferred male surgeons while 24.3% preferred female surgeons. Reputation, knowledge, and experience were the most important factors when choosing a surgeon. Male surgeons were viewed as more trustworthy, knowledgeable, experienced, and communicative. Female surgeons were dominantly perceived as more compassionate, cooperative, and prone to listen. Male respondents were 5 times more likely to choose a surgeon of similar gender (OR 5.687; CI 3.791–8.531). Male surgeons were favored for cardiovascular and orthopedic surgeries. Similarly, female surgeons were favored in gynecological and obstetric surgeries, plastic surgeries, and breast surgeries. Female gender (OR 6.193; CI 4.077–9.408), living outside Amman (OR 1.517; CI 1.066–2.160), and being married (OR 2.504; CI 1.601–3.917) were all significant positive predictors of preferring female surgeons. Our findings highlight differences in gender preference and perception of surgeons among Jordanian adults.

## Introduction

Since the establishment of the Jordanian state, gender inequality has presented itself as a major hurdle to Jordan’s national advancement^1^. The Convention on the Elimination of All Forms of Discrimination Against Women (CEDAW) reported that Jordanian women’s social status, autonomy, educational opportunities, and professional careers are undermined due to the persistence of deep-rooted discriminatory stereotypes^2^. Jordan’s social structure is a masculine-led patriarchal system. Such a system is reinforced through various mechanisms, including institutional laws providing legal ground for male dominance, limited representation in educational curricula, and media promotion of gendered stereotypes^3^. In addition, due to Jordan being an Islamic country, personal status laws are governed by the sacred Shari’a principles. However, these principles are prone to biased interpretations of religious teachings, which are a product of personal preferences and may only reflect the political and social context of their time^4^. This concerning asymmetry between genders had led females to be underrepresented in the political, economic, and employment sectors.

Throughout the years, surgical professions in Jordan have been male-dominated. Of the 26,194 doctors registered in the Jordanian Medical Counsel as of 2019, only 20.2% were female. The For Solidarity Is Global Institute (SIGI) reported that only 0.03% of Jordanian surgeons were female. Moreover, their report demonstrated that there was zero representation of females within cardiovascular surgery and neurosurgery, while only a handful of female surgeons are active within orthopedic surgery and urology. A lack of representation within leadership is also observed as only 15% of the Jordanian Medical Counsel members are female.

However, with the uprising numbers of female surgeons and residents, this gap in gender discrepancy is sealing, yet in an extremely slow fashion. Nonetheless, for these surgeons to thrive within their workplace, they must build strong bonds with their peers and patients alike^5^. Effective patient–doctor bonds, while already subject to influence by pre-existing patient preferences and gender bias, are central to providing proper care, ensuring patient satisfaction, and adherence to treatment^5,6^. Current literature mainly focuses on barriers to entry for female surgeons, lack of representation in leadership roles, lack of mentoring, and harassment^7^. In contrast, investigations about patients’ perceptions of surgeons are scant on a global scale, to say the least, and extremely rare within the Middle East.

The current body of literature demonstrates conflicting conclusions concerning patients’ gender preferences. While a myriad of studies originating from the United States show that patients do not base their choice of surgeon on gender^7,8^, others have found that both men and women are more likely to prefer a male surgeon^9,10^. Also, patients have been shown to demonstrate same-gender concordance, particularly among young patients and those with intimate concerns such as pelvic examinations^11,12^. Evidence within the Middle East, primarily conducted within Lebanon and Saudi Arabia, demonstrate similar trends of variance, yet they are primitive in terms of depth and methodological quality^13–15^.

The body of literature identifying the mechanisms behind gender discrimination showcased that the evaluation of men’s and women’s involvement in the workplace is often influenced by gender bias, a concept that finds its roots within gender stereotypes^16,17^. Gender stereotypes are defined as the shared beliefs about the attributes and abilities of both men and women^18^. Such stereotypes, regardless of their truth (or lack thereof), affect the way we perceive and evaluate others. The field of social psychology has particularly unveiled several cognitive and motivational mechanisms that lie behind gender discrimination^19^. The focus was directed toward women in employment settings where gender discrimination has been a particular problem for them, especially in male-dominant fields. Such imbalances had women at a social and economic disadvantage, as those occupations are often held in high prestige and status, which in turn, yields more monetary and social rewards^20–22^. Those fields often correspond to surgical specialties in medicine since they are highly regarded as ‘prestigious’ among the public and medical students and are often sought after because of that^23,24^.

Classic models of gender discrimination such as ‘role congruity theory’^16^, ‘lack of fit’^17,25^, and ‘think manager, think male’^26,27^ are the most evidence-supported theories that often provide consistent evidence on the psychological aspects of gender bias against women in male-dominant settings. These theories argue that a perceived mismatch between gender stereotypes and job stereotypes leads to adverse expectations for both men and women in a gender-discrepant domain, which in turn, contributes to the formation of gender discrimination.

In light of what’s above, it is essential to assess the Jordanian population’s gender preference regarding their surgeons and shed light on the reasons for possible gender bias across different surgical specialties. This will benefit female surgeons and surgical residents, as a genuine understanding of how patients perceive them will play a critical role in achieving career prosperity and acknowledgment of their abilities within the field of surgery. In addition, hospital employers will help them understand the specific needs of the population in terms of the gender of the healthcare provider, leading to better utilization and distribution of genders in each health sector to satisfy the population’s preferences.

Our study primarily aims to assess the overall gender preference of surgeons among the Jordanian population and to evaluate factors predictive of such a preference.

## Methodology

### Study design and sample size

We conducted this cross-sectional study to assess the societal preferences of Jordanian adults concerning the gender of their surgeons. Between January 20th and February 20th of 2022, Jordanian participants of both genders and different governorates were recruited, regardless of surgical history. Participants who were illiterate, under the age of 18, or did not consent to complete the questionnaire were excluded from this study. The study was facilitated through the dissemination of a self-administered survey available in both Arabic and English and in both online and print formats. Participants were randomly recruited either from public forums using social media posts or from public facilities (e.g., malls, hospitals, parks, etc.) through the distribution of a paper-based survey. The estimated sample size was calculated using GPower 3.1 and EpiInfo. At a power of 80%, α margin of error of 5%, and an effect size of 10%, a sample of 1091 participants were needed to demonstrate statistical differences of appropriate power on Chi-square.

### Constructs of gender and sex

Gender refers to a social construct addressing roles within society. On the other hand, sex refers to the biological sex given at birth. It should be noted that these two constructs, despite their differences in meaning and implications within the literature, are used interchangeably within Middle Eastern populations, particularly those of a conservative nature. Moreover, “gender” and “sex” are used interchangeably within the Arabic language. Thus, any analysis or critique of respondents’ answers should be made with consideration of the aforementioned.

### Data collection instrument

There were 19 questions in the survey, which were categorized into three domains. The questionnaire’s first domain was concerned with sociodemographic characteristics, including but not limited to age, residence, employment, income, etc. The questionnaire’s second domain investigated the following: factors affecting surgeons’ choice and characteristics associated with the preferred surgeon’s gender. The questionnaire’s final domain investigated participants’ preferences for surgeons’ gender within specific specialties. Such specialties included cardiovascular surgery, orthopedic surgery, obstetrics and gynecology, urologic surgery, plastic surgery, and surgeries involving the breast. Respondents’ answers were evaluated through the following means: (1) a four-point Likert-scale (1 = strongly disagree, 2 = disagree, 3 = agree, 4 = strongly agree), and (2) a three-point scale (1 = male, 2 = neutral, 3 = female)^28^. Omitting the midpoint (i.e., neutral) option for the Likert-scale was attempted to reduce social desirability and neutral biases^7^. Participants were also asked about their overall surgeon’s gender preference as well as their backup preference if their initial choice was unavailable (undergo surgery, change hospital, refuse the surgery)^29^.

The questionnaire was initially developed in English and then translated into Arabic by four native Arabic speakers, who were also proficient in English. An impartial expert then analyzed the questionnaire to look for double-barreled, unclear, and misleading questions. Then, a pilot test with more than 30 participants was conducted to assess readability and understandability, and test the questionnaire’s content validity and reliability. The questionnaire is provided as supplementary material (See Supplementary File 1).

### Statistical analysis

Data were analyzed using SPSS version 23. For items utilizing 4-point Likert scales, disagreement responses and agreement responses were grouped for ease of reporting. Associations between categorical variables were assessed using Chi-square. For all associations, the odds ratio (OR) and its associated confidence interval (CI) were reported. Predictors of gender preference among participants were evaluated using a multinomial multivariate regression model. Multiple correction was conducted using the Holm-Bonferroni sequential method. Correction for regression analysis was not conducted as it was an exploratory analysis of controlled predictors. Also, the conservative nature of correction reduces type I errors at the expense of increasing type II errors. Normal distribution of data was ensured by the Kolmogorov–Smirnov test of normality. All statistical tests were conducted with a 95% confidence interval and a 5% error margin. A *p*-value of less than 0.05 was considered statistically significant.

### Ethical considerations

This study was approved by the University of Jordan Institutional Review Board (No. 122/2021/0054) and followed the institutional and/or national research committee’s ethical standards and the principles of the World Medical Association’s Declaration of Helsinki. Informed consent was obtained from all participants prior to starting the questionnaire completion process. The consent form included the participants’ rights to anonymity, confidentiality of their data, and the right to leave the study. Moreover, it included reassurance that their participation is completely voluntary, is not associated with any kind of short-term benefit or reward, and does not affect the quality of their received care (if applicable).

## Results

A total of 1708 respondents were recruited in this study with a female-to-male ratio of 1.45:1. The recruited sample’s mean age was 32.9 ± 15.8 years, ranging from 18 to 96. The majority of participants (63.7%) were unmarried, had a bachelor’s degree (81.3%), and were residents of Amman (66.6%), the capital city of Jordan. In addition, 19.6% of female patients had surgery performed by a female surgeon, as opposed to 12.2% of male patients (*p*-value = 0.001). Table 1 displays the respondents' sociodemographic characteristics.

**Table 1:** Participant's sociodemographic characteristics.

Variables	Total	Male (n = 697)	Female (n = 1011)
Age (Mean ± SD) (in years)	32.0 ± 15.8	35.3 ± 17.3	31.2 ± 14.4
Marital status			
Single	1088 (63.7)	422 (60.5)	666 (65.9)
Married	620 (36.3)	275 (39.5)	345 (34.1)
Educational level			
High school or below	319 (18.7)	166 (23.8)	152 (15.1)
Bachelors or higher	1389 (81.3)	531 (76.2)	858 (84.9)
Monthly income			
Less than 500	896 (52.5)	357 (51.2)	539 (53.3)
More than 500	812 (47.5)	340 (48.8)	472 (46.7)
Residence			
Amman	1138 (66.6)	433 (62.1)	705 (69.7)
Outside amman	570 (33.4)	264 (37.9)	306 (30.3)
Employment			
Field job	357 (20.9)	171 (24.5%)	186 (18.4)
Desk job	348 (20.4)	218 (31.3)	130 (12.9)
Unemployed	852 (49.9)	247 (35.4)	605 (59.8)
Retired	151 (8.8)	61 (8.8)	90 (8.9)
Insurance			
Public sector	670 (39.2)	280 (40.2)	390 (38.6)
Private sector	569 (33.3)	221 (31.3)	348 (34.4)
None	469 (27.5)	196 (28.1)	273 (27.0)
Works or studies in the health sector			
Yes	849 (49.7)	292 (41.9)	557 (55.1)
No	859 (50.3)	405 (58.1)	454 (44.9)
Has a family member that works in the health sector			
Yes	1060 (62.1)	378 (54.2)	682 (67.5)
No	648 (37.9)	319 (45.8)	329 (32.5)
Surgery history/female surgeon			
Yes	283 (16.6)	85 (12.2)	198 (19.6)
No	1425 (83.4)	612 (87.8)	813 (80.4)
Surgery history/male surgeon			
Yes	774 (45.3)	322 (46.2)	452 (44.7)
No	934 (54.7)	375 (53.8)	559 (55.3)

Table 2 demonstrates factors affecting surgeon selection for our recruited respondents. Overall, reputation, knowledge, and experience were the most important factors when choosing a surgeon. However, male respondents were significantly less likely to consider reputation (OR: 0.591), personality (OR: 0.776), and years of experience (OR: 0.604) when choosing a surgeon compared to their female counterparts. Conversely, male respondents were significantly more likely to value family name/origins (OR: 2.082), surgical skills (OR: 1.727), and risk of surgery (OR: 1.756) when choosing their surgeon. Factors such as knowledge, religious background, and ethnicity were valued similarly by both genders of respondents, as the earlier was predominantly perceived as necessary. At the same time, the latter two were deemed not important.

**Table 2:** Factors influencing surgeon selection.

	Gender of respondents		*P*-value	Confidence interval		
	Male	Female		OR	Lower 95% CI	Upper 95% CI
Reputation			0.002	0.591	0.423	0.826
Important	617 (88.5)	939 (92.9)				
Not important	80 (11.5)	72 (7.1)				
Knowledge			0.079	0.612	0.364	1.030
Important	666 (95.6)	983 (97.2)				
Not important	31 (4.4)	28 (2.8)				
Ethnicity			0.351	1.152	0.871	1.522
Important	102 (14.6)	131 (13.0)				
Not important	595 (85.4)	880 (87.0)				
Personality			0.015	0.776	0.633	0.951
Important	438 (62.8)	693 (68.5)				
Not important	259 (37.2)	318 (31.5)				
Years of experience			0.038	0.604	0.376	0.969
Important	659 (94.5)	977 (96.6)				
Not important	38 (5.5)	34 (3.4)				
Religious background			0.065	0.790	0.619	1.009
Important	125 (17.9)	219 (21.7)				
Not important	572 (82.1)	792 (78.3)				
Family name			< 0.001	2.082	1.440	3.011
Important	72 (10.3)	53 (5.2)				
Not important	625 (89.7)	958 (94.8)				
Level of care			< 0.001	1.727	1.411	2.114
Important	293 (42.0)	299 (29.6)				
Not important	404 (58.0)	712 (70.4)				
Level of skill			< 0.001	2.070	1.688	2.539
Important	304 (43.6)	275 (27.2)				
Not important	393 (56.4)	736 (72.8)				
Risk of surgery			< 0.001	1.756	1.438	2.144
Important	311 (44.6)	318 (31.5)				
Not important	386 (55.4)	693 (68.5)				

When asked to associate a set of unique characteristics with surgeons’ gender, participants were dominantly neutral. For the exception of compassion, which showed a greater propensity towards choosing females (46.1%), characteristics such as trustworthiness (66.8%), knowledge (67.4%), experience (62.2%), communication skills (53.6%), cooperation (59.5%), and listening skills (51.9%) were dominated by neutral responses.

When analyzing only those with a gender preference, participants of both genders concur that male surgeons are more trustworthy, knowledgeable, experienced, and communicative. On the other hand, female surgeons were dominantly perceived as more compassionate, cooperative, and prone to listening. Our results demonstrate that when asked if the surgeon’s gender is associated with unique positive characteristics, respondents of either gender associated good surgeon characteristics with their gender. Both male and female respondents were significantly more likely to attribute characteristics such as trustworthiness (OR: 1.952), compassion (OR: 2.161), knowledge (OR: 1.669), and proper communication skills (OR: 1.561) to their gender. Our data show that both sets of respondents’ overall preferences are significantly different (< 0.001). Male respondents are 5 times more likely to choose a surgeon of a similar gender (OR: 5.687). Overall, irrespective of respondents’ gender, male was the preferred gender of surgeons. It should be noted that when including participants with neutral responses, the overall preference of surgeons’ gender was neutral (52.0%) compared to 36.3% preferring males and 11.7% preferring females. Table 3 shows which gender was associated with unique positive characteristics. Interestingly, 15.3% and 20.5% of female and male participants were willing to cancel their emergency surgeries or change hospitals if their preferred gender of surgeons was not available.

**Table 3:** The association of certain positive characteristics with surgeon’s gender.

	Gender of respondents		*P*-value	OR	Confidence interval	
	Male	Female			Lower 95% CI	Upper 95% CI
Trustworthiness			< 0.001	1.952	1.397	2.728
Male	181 (61.4)	122 (44.9)				
Female	114 (38.6)	150 (55.1)				
Compassion			< 0.001	2.161	1.591	2.935
Male	127 (28.9)	89 (15.8)				
Female	313 (71.1)	474 (84.2)				
Knowledge			0.011	1.669	1.132	2.461
Male	235 (79.7)	183 (70.1)				
Female	60 (20.3)	78 (29.9)				
Experience			0.251	1.333	0.849	2.092
Male	300 (88.0)	258 (84.6)				
Female	41 (12.0)	47 (15.4)				
Communication skills			0.002	1.561	1.179	2.068
Male	214 (57.4)	194 (46.3)				
Female	159 (42.6)	225 (53.7)				
Cooperation			0.939	1.018	0.755	1.373
Male	151 (45.9)	165 (45.5)				
Female	178 (54.1)	198 (54.5)				
Listening skills			0.344	0.869	0.652	1.157
Male	131 (34.2)	164 (37.4)				
Female	252 (65.8)	274 (62.6)				
Overall preference			< 0.001	5.687	3.791	8.531
Male	329 (90.9)	291 (63.7)				
Female	33 (9.1)	166 (36.3)				

When asked about surgeons' gender preference per type of surgery, respondents barely had any notable differences. Male surgeons were heavily favored by respondents of both genders for cardiovascular and orthopedic surgeries. Similarly, female surgeons were heavily favored in gynecological and obstetric surgeries, plastic surgeries, and breast surgeries (refer to Table 4). However, when asked about urologic surgeries, both respondents were 12 times significantly more likely to choose a surgeon of their same gender (OR: 12.547).

**Table 4:** Gender presence of surgeons per specific types of surgery.

	Gender of respondents		*P* value	OR	Confidence interval	
	Male	Female			Lower 95% CI	Upper 95% CI
Cardiovascular surgery			0.099	1.473	0.941	2.304
Male	394 (92.5)	510 (89.3)				
Female	32 (7.5)	61 (10.7)				
Orthopedic surgery						
Male	432 (92.5)	582 (91.7)	0.654	1.124	0.721	1.753
Female	35 (7.5)	53 (8.3)				
Obstetrics and gynecology			0.504	1.124	0.812	1.555
Male	72 (14.3)	102 (12.9)				
Female	431 (85.7)	686 (87.1)				
Urologic surgery			< 0.001	12.547	9.032	17.429
Male	394 (88.1)	237 (37.2)				
Female	53 (11.9)	400 (62.8)				
Surgery involving the breast			0.570	1.084	0.821	1.431
Male	109 (22.9)	155 (21.6)				
Female	366 (77.1)	564 (78.4)				
Plastic surgery			0.356	0.878	0.677	1.137
Male	171 (43.4)	263 (46.6)				
Female	223 (56.6)	301 (53.4)				

### Predictors of preference of female surgeons compared to males

In terms of factors predictive of preference for female surgeons over males, a multinomial regression model demonstrated the following: Female gender (OR: 6.193; *p* < 0.001; 95% CI 4.077–9.408), living outside Amman (OR: 1.517; *p*: 0.021; 95% CI 1.066–2.160), and being married (OR: 2.504; *p* < 0.001; 95% CI 1.601–3.917) were all significant positive predictors of preferring female surgeons over male surgeons. All other factors, such as educational level, income level, and age among others, were insignificant (Refer to Table 5).

**Table 5:** Multinomial regression of factors predictive of female gender preference of surgeons in reference to male surgeons.

Variable	*P* value	OR	Lower 95% CI	Upper 95% CI
Age	0.896	1.001	0.987	1.016
Level of education				
Higher education	0.725	0.925	0.601	1.424
Primary education (REF)				
Income level				
< 500 JDs	0.788	0.951	0.657	1.376
> 500 JDs (REF)				
Insurance type				
Public Ins	0.613	0.898	0.592	1.362
Private Ins	0.826	0.953	0.619	1.467
No Ins (REF)				
Works in healthcare				
Yes	0.228	0.769	0.502	1.178
No (REF)				
Family member in healthcare				
Yes	0.484	0.880	0.615	1.258
No (REF)				
Marital status				
Married	0.000	2.504	1.601	3.917
Single (REF)				
Residence				
Outside amman	0.021	1.517	1.066	2.160
Amman (REF)				
Gender				
Female	0.000	6.193	4.077	9.408
Male (REF)				

### Predictors of gender preference in reference to neutral

Another multinomial regression model was implemented to investigate the predictors of surgeons gender preference among recruited participants in reference to being neutral (See Supplementary Table S1). Among those working in healthcare occupations, being male was associated with a higher likelihood of preferring male surgeons (OR: 3.060; *p* < 0.001; 95% CI 2.213–4.232). By the same token, male gender was a negative predictor of preferring female surgeons (OR: 0.142; *p* < 0.001; 95% CI 0.056–0.358). On the other hand, for those who are not working in healthcare fields, older age was a weak positive predictor of preferring male surgeons (OR: 1.025; *p* < 0.001; 95% CI 1.013–1.037). Conversely, being single (OR: 0.599;* p*: 0.005; 95% CI 0.420–0.854) and having a degree in higher education (OR: 0.582; *p*: 0.004; 95% CI 0.401–0.844) predicted a negative preference towards male surgeons. Finally, for participants preferring female surgeons over not having a preference, age, gender, a degree in higher education, and having a family member in healthcare were significant predictors of such preference.

## Discussion

In this survey of Jordanian adults, we demonstrated that about half of the recruited participants have portrayed no preference for their surgeon’s gender. These results are in tandem with the literature, as several studies have shown that various populations display a dominant propensity not to have a gender preference^5,7,8,13,29^. Evidence shows that people’s preference for surgeons is dependent on professional qualifications (e.g., demeanor and competence) and communication skills rather than personal characteristics such as gender^7,30^. The notion of neutrality towards surgeons’ gender among Jordanians could also be attributed to other factors. Firstly, participants, especially younger adults, may demonstrate social desirability bias within their responses, which favors a lack of preference. Secondly, it is hypothesized that personal or familial history with surgical procedures may impact overall preference^30^.

Nonetheless, among those who did demonstrate a preference, gender preference was strongly dominated towards male surgeons. In fact, males were significantly more likely to prefer male surgeons than their female counterparts, who also preferred male surgeons. An investigation of 3015 Saudi adults demonstrated that the same gender preference of surgeons exhibited by males is attributed to the fact that male respondents perceived male surgeons as more trustworthy, compassionate, knowledgeable, and having better communication skills^31^; a finding mostly consistent with our results. These findings were also true for a survey of 1000 Lebanese adults, among whom, those who preferred male surgeons, perceived them as more trustworthy, brave, and competent^13^. On the other hand, studies show that females are often neutral when it comes to gender preference^5,31,32^; this may stem from the prioritization of other physician attributes when the choice of surgeon is on the table. Nonetheless, females are more likely to choose same-gendered physicians when their concerns are either intimate or when they seek empathy, which is associated with the female-to-female dyad style of conversation. It should be noted that studies conducted on Middle Eastern populations demonstrate far greater rates of gender preference compared to their Western counterparts, which may imply cultural and economic components to this variance^33^.

Among our participants, female surgeons were perceived as more compassionate and cooperative, and as better listeners than their male counterparts. In support of this finding, a meta-analysis concluded that female physicians are often patient-centered with their communication and employ higher levels of emotional talk than their male peers^34^. Moreover, evidence demonstrates that female physicians are more nurturing and sensitive than males, as they make effective use of skills such as empathy, sympathy, and concern^34,35^.

Gender stereotypes are established and further bolstered through the differential allocation of roles and occupations in society. These gender-type beliefs for different occupations emerge very early and are often difficult to conceal^36,37^. Men are often portrayed as agentic (i.e., associated with attributes of assertiveness, achievement orientation, and autonomy) because of their historical dominance in income provision roles and occupations that are associated with power. Women, on the other hand, are mostly aligned with attributes of communality (i.e., being emotionally sensitive and highly considerate of others) because of their overrepresentation in caregiving occupations^38,39^. These stereotypes are composed of descriptive (what characterizes each gender group, i.e., associating women with emotionality and men with rationality) and perspective (women ought to be caring, men should be strong) components^40,41^. Discrimination often arises when descriptive stereotypes assume a differential perception of men’s and women’s competence in specific occupations. Perspective stereotypes, on the other hand, lead to discrimination through the social penalization of those who violate societal norms^17,42^. The aforementioned descriptive stereotypes are not always associated with negative outcomes. However, when these stereotypes are at odds with what is thought to predict success in specific occupations, gender discrimination arises.

Gender isolated, our participants declared that the surgeon’s medical knowledge, experience, and reputation are the most impactful factors when choosing a surgeon. Although other factors such as board certification and interpersonal skills were pronounced necessary, our results remain consistent with the literature for the most part^14,43^. On the other hand, ethnicity, religion, and family name/origin were the least likely to influence our participants' decisions when choosing a surgeon. Whether religion impacts the choice of surgeon is a matter of controversy. Saudi women at the King Fahd General Hospital and the Maternity and Children Hospital deemed religion a vital factor in their choice of either surgeon or gynecologist^15^. In contrast, for Saudi women surveyed at the King Abdulaziz University Hospital, the religion of participants did not impact their preference for their gynecologist’s gender^14^. Moreover, religion played no role in the choice of orthopedic surgeons in the United States^8^. While the lack of association does support our previous conclusions, especially among females, the presence of a correlation between religion and the preference of physician may be attributed to the patient’s desire for commonality, by which same-religion physicians are perceived as more apt in understanding factors influencing treatment decisions and in providing more comfortable communication^8,44^. Therefore, this dispute could possibly be attributed to the population’s view on religious diversity and coexistence or the surgeon’s specialty.

Despite our results showing an overall preference for male surgeons, deviations were apparent when the surgeon’s specialty was particularly identified. Participants preferred male surgeons in cardiovascular and orthopedic surgeries, thus suggesting that there exists a gender bias for males in high-risk scenarios. This begs the question, “Does increased representation improve the quality of care within certain specialties, or do historical stereotypes feel more comfortable to patients?”. Moreover, despite the considerable progress worldwide and in Jordan in particular, orthopedic surgery remains a male-dominated field and is amongst the least desired programs by female physicians. This stereotype could have refrained people from believing women could pursue that role due to its physical demand or the lack of female role models in the field^45^. Pointing out exactly whether male preference in such specialties is due to a real difference in skill or a mere chronic effect of historic misrepresentation is conflicting even within the literature, as some studies reported no gender preference for orthopedic surgeons, while others demonstrated a preference for same-gendered surgeons, especially among males^8,13,31^.

Interestingly, when asked about gender preference in urologic surgery, male respondents had a significant preference for males and female respondents for females. This patient-surgeon same-gender concordance was in uniform with the literature^31,46,47^. A possible reason behind this phenomenon could be the greater status congruence achieved when patients receive medical attention from same-gender physicians. This augments mutual participation and establishes a framework for collaborative decision-making and self-disclosure during the surgical consultation. Therefore, more promising outcomes are expected. Moreover, female respondents preferred female surgeons to perform obstetric, gynecological, and breast surgeries. The same preference was also demonstrated by male participants. The earlier can be explained by the fact that most women in Muslim countries, and Jordan is no exception, prefer having a female gynecologist to abide by the religious belief that mandates that in certain cases, such as that of medical treatment, only a female is authorized to view, examine, and treat a woman’s sensitive areas, which include the breast and entire pelvis^48^. Moreover, the Western literature shows that females would prefer a same-gendered practitioner if their concerns are intimate, such as those relating to genital pathologies^32^. Studies in Saudi Arabia also reported that the additional influence of society, comfort, and husbands' preferences may account for this demeanour^14,15^, which may explain the latter observation as males are culturally appointed as ultimate caretakers in patriarchal societies such as that of the Middle East. Thus, this exclusive preference should be expected within countries of similar cultural backgrounds and social structures.

Within our sample, 15.3% of females demonstrated extreme rejection of the undesirable surgeon’s gender. They stated that in cases of emergency where their preferred surgeon's gender was unavailable, they would change hospitals or not undergo surgery. Although an appalling indicator, this comes as no surprise as Alsubyani et al. reported a comparable value of 11.6% among Saudi women^15^. When patients are willing to put their lives at risk purely for the sake of the availability of their preferred surgeon's gender, one could confidently recommend immediate policy changes to tackle such a phenomenon. This is an exemplary showcasing of how gender differences impact physician–patient interactions, expectations, and degrees of equivalence^49^. Thus, to satisfy the needs of a diverse patient population, diverse health workforce representation is necessary, including but not limited to gender representation. As diversity in the medical field continues to grow, a more profound emphasis on health-seeking behavior is essential to building a clinical field devoid of disparities and ultimately fostering optimal health outcomes. This could be achieved through training surgical residents on proper communication skills and building healthy surgeon–patient relations to ensure equal clinical care for both genders.

Despite females forming about half of the health workforce in Jordan, they continue to be underrepresented and have a limited role in senior healthcare decision-making. This shortage of women in line management can be postulated to the persistent traditional gender norms, poor enforcement of policies, childbearing, domestic responsibilities, and discrimination in recruitment^50^. In addition, gender-based discrimination in the healthcare environment impedes the advancement of females’ careers and further exacerbates their underrepresentation. Women are subjected to gender bias in several forms, such as exclusion from opportunities, disrespect, sexual harassment, authority abuse, and unequal expectations^51^.

Gender disparities are part of institutional conscious or unconscious preferences, biased systematically against females in various professions. Conscious bias exists within a person’s entire perception, hence reflecting on one’s behavior. Conversely, unconscious bias is defined as unintentional associations based on gender, traditions, culture, society, and experiences^52^. For example, the inequitable male and female availability in specific surgical fields is likely to compromise patient-oriented outcomes (e.g., perceived quality of care and satisfaction); moreover, such disparity might be a result of the aforementioned biases^53,54^. In addition, some female patients favor female surgeons and therefore reflect on the patients' contentment and health. An analysis of the Gender-Career IAT project demonstrated that females face strong implicit and explicit biases as they are strongly associated with family and not career, and in the case of female physicians, they are strongly associated with family medicine and not surgery^55^. This raises an urgent call to root out gender biases in our society and promote a general understanding of gender-related challenges. To address gender inequality, institutional legislators should implement gender-neutral recruitment processes, standardize income, encourage female mentorship programs, have a transparent policy on gender discrimination, actively support women in more senior roles, and promote meritocracy^52,56^.

The investigation of gender preferences of people towards surgeons is an understudied topic worldwide and has been overlooked in our region. This is the first study to analyze patients’ preferences for surgeons’ gender among a relatively large and powerful sample of Jordanian adults. Nonetheless, our conclusions are limited by the following: technology bias as the sample consisted mainly of young people with bachelor’s degrees living in Amman, which is the sector of the population with the greatest internet access. This could have undermined the influence of the elderly, the uneducated, and those living in other Jordanian governorates. In addition, participants were restricted to closed-ended answers, preventing them from giving in-depth insights. Moreover, for participants that expressed gender preference, they were not able to justify their choice, thus laying a massive emphasis on performing future qualitative studies to assess potential reasoning behind their preferences and investigate more influential surgeons' characteristics—ultimately providing more information on improving the gender gap across various surgical specialties.

## Conclusion

While the percentage of participants with no gender preference is acceptable, about half of our sample had a gender preference, mostly toward male surgeons. Considering the probable risk inflicted on the health of patients with such preferences, guaranteeing the availability of surgeons who meet patients’ desires may prove to be crucial. Hence, healthcare administrations should direct more efforts toward educating the public, identifying gender bias, and resolving any form of gender-based misconceptions.

## Supplementary Information

Supplementary Information.

Supplementary Table 1.

## Data Availability

The datasets used and/or analysed during the current study are available from the corresponding author on reasonable request.
