# Supplementary material for: Factors influencing gender preference towards surgeons among Jordanian adults: an investigation of healthcare bias
Source: Sci Rep. 2023 Jul 18;13:11614. doi: 10.1038/s41598-023-38734-1 (PMC10354224; doi:10.1038/s41598-023-38734-1)
Supplement: Supplementary file 1 — Supplementary Information. [file 41598_2023_38734_MOESM1_ESM.docx]

**The Questionnaire – Supplementary material**

*The questionnaire was shared in the Arabic language (inserted below).*

**Factors influencing gender preference towards surgeons among Jordanian adults: an investigation of healthcare bias**

This is a cross-sectional study done by medical students at the university of Jordan under the supervision of Dr. Abdallah Al-Ani. The study aims to measure the preference of the Jordanian population towards the surgeon’s gender. Your participation is highly appreciated!

No personally identifying information will be required, and data will be handled with confidentiality. No direct benefits will be offered, however your responses will help us better understand disparities in healthcare on the long term.

If you have any questions regarding this study, you are welcome to contact the research team at any time through the following email: [ramarayyan@gmail.com](mailto:ramarayyan@gmail.com).

**Do you agree to participate in this study? Yes/No**

**Demographics**

1. Gender
   1. Female
   2. Male
2. Age?
3. Marital Status
   1. Single
   2. Married
   3. Divorced/Widowed/Separated
4. Educational Level
   1. Primary School
   2. Middle School
   3. Secondary School
   4. Bachelors/College or Higher
5. Monthly Income
   1. 0-150 JDs
   2. 150-500 JDs
   3. 500-1000 JDs
   4. More than 1000 JDs
6. Residence
   1. Amman
   2. Outside Amman
7. Employment
   1. Field Job
   2. Desk Job
   3. Unemployed
   4. Retired
8. Type of insurance
9. Public sector
10. Private sector
11. Non
12. Have you ever worked or studied in the health sector? Yes/No
13. Do you have any family member working or studying in the health sector? Yes/No

**General**

1. How do you evaluate the following factors affecting your surgeon selection?

|  | Very important | Important | Slightly important | Not important |
| --- | --- | --- | --- | --- |
| - 1. Reputation |  |  |  |  |
| - 1. Knowledge |  |  |  |  |
| - 1. Ethnicity |  |  |  |  |
| - 1. Personality |  |  |  |  |
| - 1. Experience |  |  |  |  |
| - 1. Religion |  |  |  |  |
| - 1. Family name |  |  |  |  |

1. Have you ever performed surgery under female provision? Yes/No
2. Have you ever performed surgery under male provision? Yes/No
3. Do you believe that the surgeon’s gender affects the level of your care?

(Strongly agree/Agree/Disagree/Strongly disagree)

1. Do you believe that the surgeon’s gender affects their surgical skills?

(Strongly agree/Agree/Disagree/Strongly disagree)

1. Evaluate surgeon’s performance in the following aspects:^1^

|  | Male | Female | Doesn’t matter |
| --- | --- | --- | --- |
| - 1. Trustworthy |  |  |  |
| - 1. Compassion |  |  |  |
| - 1. Knowledge |  |  |  |
| - 1. Experience |  |  |  |
| - 1. Communication skills |  |  |  |
| - 1. Cooperative |  |  |  |
| - 1. Listening skills |  |  |  |

1. Overall surgeon gender preference?
   1. Female
   2. Male
   3. Neutral
2. In case of emergency, if your preference surgeon gender is not available what decision will you take?^2^
   1. Undergo the surgery
   2. Change hospital
   3. Will not do it

**Type of surgery**

1. Do you believe that the risk of surgery affects your gender of choice?

(Strongly agree/Agree/Disagree/Strongly disagree)

1. Select the preferred surgeon’s gender for the following surgeries:

|  | Female | Male | Neutral |
| --- | --- | --- | --- |
| - 1. Orthopaedic surgery |  |  |  |
| - 1. Obstetrics and gynecology surgery |  |  |  |
| - 1. Cardiovascular surgery |  |  |  |
| - 1. Urologic surgery |  |  |  |
| - 1. Plastic surgery |  |  |  |
| - 1. Breast surgery |  |  |  |

**References**

1. Alyahya G, Almohanna H, Alyahya A, et al. Does physicians’ gender have any influence on patients’ choice of their treating physicians? *J Nat Sci Med*. 2019;2(1):29.

2. Alsubyani NA, Almassri RT, Alnasr LM, Mitha SS, Albloshi LA. The prevalence of surgeon gender preference for female patients in surgery and ob/gyn. In: *Proceedings of Research Fora, International Conference, Dubai, United Arab Emirates*. ; 2017:2-3.
