# Supplementary material for: Factors influencing gender preference towards surgeons among Jordanian adults: an investigation of healthcare bias
Source: Sci Rep. 2023 Jul 18;13:11614. doi: 10.1038/s41598-023-38734-1 (PMC10354224; doi:10.1038/s41598-023-38734-1)
Supplement: Supplementary file 2 — Supplementary Table 1. [file 41598_2023_38734_MOESM2_ESM.docx]

**Factors influencing gender preference towards surgeons among Jordanian adults: an investigation of healthcare bias**

Supplementary table 1:

| **Works in Health section** | **Overall preference** | **Variable** | **Sig.** | **OR** | **Lower 95%CI** | **Upper 95%CI** |
| --- | --- | --- | --- | --- | --- | --- |
| Yes | Male | **Age** | 0.081 | 1.021 | 0.997 | 1.045 |
|  |  | **Gender** |  |  |  |  |
|  |  | Male | **0.000** | 3.060 | 2.213 | 4.232 |
|  |  | Female (REF) | - |  |  |  |
|  |  | **Marital Status** |  |  |  |  |
|  |  | Single | 0.202 | 0.632 | 0.313 | 1.279 |
|  |  | Married (REF) |  |  |  |  |
|  |  | **Education** |  |  |  |  |
|  |  | Higher education | 0.126 | 1.545 | 0.885 | 2.697 |
|  |  | Primary education (REF) |  |  |  |  |
|  |  | **Monthly Income** |  |  |  |  |
|  |  | < 500 JDs | 0.231 | 1.262 | 0.862 | 1.848 |
|  |  | > 500 JDs (REF) |  |  |  |  |
|  |  | **Residence** |  |  |  |  |
|  |  | Amman | 0.912 | 0.980 | 0.682 | 1.407 |
|  |  | Outside Amman (REF) |  |  |  |  |
|  |  | **Insurance type** |  |  |  |  |
|  |  | Public Ins | 0.313 | 1.245 | 0.813 | 1.907 |
|  |  | Private Ins | 0.287 | 1.285 | 0.810 | 2.039 |
|  |  | No Ins (REF) |  |  |  |  |
|  |  | **Meh** |  |  |  |  |
|  |  | Yes | 0.814 | 1.045 | 0.723 | 1.510 |
|  |  | No |  |  |  |  |
|  | Female | **Age** | 0.801 | 0.994 | 0.948 | 1.042 |
|  |  | **Gender** |  |  |  |  |
|  |  | Male | 0.000 | 0.142 | 0.056 | 0.358 |
|  |  | Female (REF) |  |  |  |  |
|  |  | **Marital Status** |  |  |  |  |
|  |  | Single | 0.954 | 0.964 | 0.276 | 3.363 |
|  |  | Married (REF) |  |  |  |  |
|  |  | **Education** |  |  |  |  |
|  |  | Higher education | 0.916 | 1.040 | 0.503 | 2.152 |
|  |  | Primary education (REF) |  |  |  |  |
|  |  | **Monthly Income** |  |  |  |  |
|  |  | < 500 JDs | 0.671 | 1.127 | 0.650 | 1.952 |
|  |  | > 500 JDs (REF) |  |  |  |  |
|  |  | **Residence** |  |  |  |  |
|  |  | Amman | 0.335 | 0.784 | 0.478 | 1.285 |
|  |  | Outside Amman (REF) |  |  |  |  |
|  |  | **Insurance type** |  |  |  |  |
|  |  | Public Ins | 0.510 | 0.827 | 0.469 | 1.456 |
|  |  | Private Ins | 0.296 | 0.713 | 0.378 | 1.345 |
|  |  | No Ins (REF) |  |  |  |  |
|  |  | **Meh** |  |  |  |  |
|  |  | Yes | 0.199 | 1.433 | 0.828 | 2.478 |
|  |  | No |  |  |  |  |
| No | Male | **Age** | 0.000 | 1.025 | 1.013 | 1.037 |
|  |  | **Gender** |  |  |  |  |
|  |  | Male | 0.712 | 0.943 | 0.690 | 1.288 |
|  |  | Female (REF) |  |  |  |  |
|  |  | **Marital Status** |  |  |  |  |
|  |  | Single | 0.005 | 0.599 | 0.420 | 0.854 |
|  |  | Married (REF) |  |  |  |  |
|  |  | **Education** |  |  |  |  |
|  |  | Higher education | 0.004 | 0.582 | 0.401 | 0.844 |
|  |  | Primary education (REF) |  |  |  |  |
|  |  | **Monthly Income** |  |  |  |  |
|  |  | < 500 JDs | 0.146 | 1.323 | 0.907 | 1.929 |
|  |  | > 500 JDs (REF) |  |  |  |  |
|  |  | **Residence** |  |  |  |  |
|  |  | Amman | 0.864 | 0.972 | 0.702 | 1.345 |
|  |  | Outside Amman (REF) |  |  |  |  |
|  |  | **Insurance type** |  |  |  |  |
|  |  | Public Ins | 0.537 | 0.887 | 0.606 | 1.298 |
|  |  | Private Ins | 0.208 | 0.789 | 0.545 | 1.141 |
|  |  | No Ins (REF) |  |  |  |  |
|  |  | **Family members in healthcare** |  |  |  |  |
|  |  | Yes | 0.204 | 0.812 | 0.589 | 1.120 |
|  |  | No |  |  |  |  |
|  | Female | **Age** | 0.001 | 1.027 | 1.011 | 1.044 |
|  |  | **Gender** |  |  |  |  |
|  |  | Male | 0.000 | 0.264 | 0.160 | 0.437 |
|  |  | Female (REF) |  |  |  |  |
|  |  | **Marital Status** |  |  |  |  |
|  |  | Single | 0.119 | 1.478 | 0.904 | 2.215 |
|  |  | Married (REF) |  |  |  |  |
|  |  | **Education** |  |  |  |  |
|  |  | Higher education | 0.047 | 0.580 | 0.339 | 0.993 |
|  |  | Primary education (REF) |  |  |  |  |
|  |  | **Monthly Income** |  |  |  |  |
|  |  | < 500 JDs | 0.874 | 1.046 | 0.603 | 1.813 |
|  |  | > 500 JDs (REF) |  |  |  |  |
|  |  | **Residence** |  |  |  |  |
|  |  | Amman | 0.072 | 0.648 | 0.403 | 1.040 |
|  |  | Outside Amman (REF) |  |  |  |  |
|  |  | **Insurance type** |  |  |  |  |
|  |  | Public Ins | 0.922 | 0.972 | 0.551 | 1.714 |
|  |  | Private Ins | 0.792 | 0.929 | 0.535 | 1.613 |
|  |  | No Ins (REF) |  |  |  |  |
|  |  | **Family members in healthcare** |  |  |  |  |
|  |  | Yes | 0.046 | 0.616 | 0.383 | 0.991 |
|  |  | No |  |  |  |  |
